# Acrylamide- and Hydroxymethylfurfural-Forming Capacity of Alternative Flours in Heated Dough Systems †

**DOI:** 10.3390/foods14091597

**Published:** 2025-04-30

**Authors:** Marta Mesias, Francisco J. Morales

**Affiliations:** Institute of Food Science, Technology and Nutrition (ICTAN), Spanish National Research Council (CSIC), 28040 Madrid, Spain; fjmorales@ictan.csic.es

**Keywords:** acrylamide, flour, cereal-based foods, baking, risk

## Abstract

The use of alternative flours is becoming more common in the food industry to enhance the nutritional and sensory properties of baked goods. However, these changes may also affect the formation of acrylamide, a potentially carcinogenic and genotoxic compound generated in foods heated above 120 °C. This study evaluated the acrylamide-forming potential of 16 flours from cereals, pseudocereals, legumes, fruits, and roots. Samples were analyzed for acrylamide precursors—reducing sugars and free asparagine—and tested in model dough systems with and without added glucose. All samples were baked at 150 °C for 30 min. Hydroxymethylfurfural (HMF) was also determined as a marker of thermal damage. In water-hydrated systems, acrylamide was only detected in wheat, rye, and coconut flours (23–61 µg/kg). When glucose was added, acrylamide levels increased in all systems except cassava. Lentil flour produced the highest levels (154 µg/kg), while corn flour showed the lowest (20 µg/kg). HMF levels followed a similar trend, with lentil flour again showing the highest content (232.3 mg/kg). These results highlight the importance of evaluating acrylamide formation when using non-wheat flours, especially in formulations containing sugars. Additional mitigation strategies may be needed to ensure the safety of these innovative food products.

## 1. Introduction

The growing global demand for healthier and more sustainable food options has driven the exploration of alternative ingredients to replace conventional cereal grains in food production. Minor cereals, pseudocereals, legumes, seeds, fruits and roots are attracting increasing attention due to their nutritional benefits, distinctive functional properties, and potential to enhance the sustainability of food systems [1,2]. Their integration into the bakery industry has enabled the development of a wide variety of baked goods, many of which are suitable for consumers with gluten intolerance and celiac disease, while also providing additional nutritional advantages [3]. Nevertheless, the application of these novel ingredients in baking, particularly within cereal-based products, remains less studied compared to traditional cereals like wheat, especially regarding the formation of harmful compounds during thermal processing. Baking typically involves exposing the food product to high temperatures, which can reach up to 260 °C [4]. These elevated temperatures, coupled with the low moisture content typical of cereal-based products, promote the Maillard reaction. This reaction improves the textural and sensory characteristics of baked goods by enhancing flavors, colors, and aromas that are highly appreciated by consumers. However, it can also lead to undesirable outcomes, such as the natural formation of chemical contaminants like acrylamide and hydroxymethylfurfural (HMF) [5,6].

Acrylamide is a chemical compound classified as a probable human carcinogen by the International Agency for Research on Cancer (IARC) [7]. It is naturally formed in foods during high-temperature processing, typically above 120 °C, through the Maillard reaction between reducing sugars and the free amino acid asparagine [8]. Studies have shown that acrylamide can cause genetic mutations and damage to the nervous system in animal models. Although the evidence in humans is still limited, its potential genotoxicity and carcinogenicity have raised significant concerns regarding long-term dietary exposure, prompting health authorities to recommend strategies to reduce its presence in food.

Numerous studies have documented acrylamide formation in foods subjected to thermal processing methods such as frying, baking, and roasting, including products like potato chips and French fries [6], vegetable crisps [9], breaded foods [10], bakery products, roasted nuts [11], and coffee [12]. In response to this issue, the European Food Safety Authority (EFSA) identified acrylamide in food as a public health concern [8]. This led to the implementation of European Commission Regulation (EU) 2017/2158, which established mitigation measures and benchmark levels for acrylamide reduction, particularly in foods such as potato products, cereal-based items, coffee, and coffee substitutes [13]. A subsequent recommendation in 2019 expanded monitoring to include additional food categories [14]. In parallel, 5-hydroxymethylfurfural (HMF) is formed as an intermediate product of the Maillard reaction and through the thermal degradation of sugars at elevated temperatures, serving as a useful indicator of heat-induced food damage. Research in animal models has shown that HMF can be metabolized into 5-sulfoxymethylfurfural, a compound with confirmed genotoxic and mutagenic properties [15,16]. Given the potential health risks posed by both acrylamide and HMF, it is essential that we explore and implement effective mitigation strategies to reduce their formation during food processing and enhance consumer safety.

In addition to being a source of exposure to HMF [17], cereals represent a major source of acrylamide intake in the human diet, with the asparagine content playing a critical role in the formation of this contaminant [5]. Therefore, implementing strategies to reduce acrylamide formation across the entire cereal production chain—from cultivation to consumption—is crucial for ensuring food safety. Effective mitigation can be achieved by carefully managing the levels of precursors in raw materials and selecting recipe ingredients thoughtfully. While significant research has focused on acrylamide formation in traditional cereals like wheat, rice, and corn [5,13,18], studies on minor cereals, pseudocereals, and other alternative ingredients used to replace wheat in cereal-based products are still limited. This gap in knowledge highlights the need to explore the potential of these alternative ingredients in addressing acrylamide formation during food processing.

This paper aimed to explore the formation of acrylamide during the baking process of flours derived from minor cereals, pseudocereals, legumes, fruits and roots, which are commonly used in the food industry. Furthermore, it examined the impact of flour type and the addition of glucose to a dough model system on browning development and acrylamide formation during baking. The formation of HMF was also evaluated as an indicator of thermal processing.

## 2. Materials and Methods

### 2.1. Chemicals

All chemicals and reagents used were of analytical grade. Acrylamide isotopically labelled with ^13^C_3_ (99%) was obtained from Cambridge Isotope Laboratories (Andover, MA, USA). Formic acid (98%) and methanol (99.5%) were supplied by Panreac (Barcelona, Spain). Deionized water for all solution preparations was sourced from a Milli-Q Integral 5 water purification system (Millipore, Billerica, MA, USA). All other reagents were purchased from Sigma-Aldrich (St. Louis, MO, USA).

### 2.2. Samples

Flours derived from whole grains, pseudocereals, legumes, fruits, and roots were obtained from a local food store and sourced from four different commercial brands. These included wheat (*Triticum aestivum*) as traditional cereal, minor cereals—corn (*Zea mays*), durum wheat (*Triticum durum*), oat (*Avena sativa*), rice (*Oryza sativa*), rye (*Secale cereale*), and spelt (*Triticum spelta*); pseudocereals—buckwheat (*Fagopyrum esculentum*), quinoa (*Chenopodium quinoa*), and teff (*Eragrostis tef*); legumes—chickpea (*Cicer arietinum*), lentils (*Lens culinaris*), soybean (*Glycine max*); fruits—chesnut (*Castanea sativa*), coconut (*Cocos nucifera*), and roots—cassava (*Manihot esculenta*). A 0.5 g portion of each flour was transferred into screw-cap Pyrex tubes. Two milliliters of deionized water and 1 mL of sodium chloride solution (5 mg/mL) were added to the samples. The mixture was vortexed for 5 min, left to stand at room temperature for 30 min, and then centrifuged at mild conditions (1400× g) for 10 min. The supernatant was discarded, and the tubes containing the dough with retained water were sealed and baked at 150 °C for 30 min in a forced-air convection oven (Memmert UNE 400, Schwabach, Germany). These samples were referred to as water-hydrated systems. For each type of flour, eight repetitions were prepared and grouped into two replicates. Additional experiments were conducted by substituting 2 mL of water with 2 mL of glucose solution (0.15 g/mL), along with 1 mL of sodium chloride solution (5 mg/mL). These samples were referred to as glucose-hydrated systems. Similarly, eight repetitions per flour type were prepared and combined into two replicates of four samples each.

### 2.3. Determination of Moisture

The moisture content of the flours was determined gravimetrically by drying to a constant weight in an oven at 105 °C for 24 h, following the Association of Official Analytical Chemists (AOAC) method [19].

### 2.4. Determination of Water-Holding Capacity (WHC)

Five grams of flour were placed into a pre-weighed centrifuge tube, and 30 mL of water was added. The mixture was vigorously vortexed for 1 min, left to stand at room temperature for 30 min, and centrifuged at 1400× *g* for 15 min. After centrifugation, the unretained water was discarded, and the tube was weighed. The water-holding capacity (WHC) of the samples was calculated using the following formula:

(1) $$\mathrm{WHC}=(\frac{weight\;of\;tube\;with\;sample\;and\;retained\;water-weight\;of\;tube\;with\;sample}{\mathrm{weight}\;\mathrm{of}\;\mathrm{sample}})\times 100$$

Results are expressed as g of water retained/100 g of flour (%).

### 2.5. Determination of pH

Samples of flour or systems hydrated with water or glucose (0.25 g) were mixed with 25 mL of water and vortexed for 3 min. The mixture was then left to stand at room temperature for 1 h before being centrifuged to separate the phases. The pH of the supernatant was measured using a CG-837 pH meter (Schott, Mainz, Germany).

### 2.6. Determination of Color

Color measurements were performed using a HunterLab Spectrophotometer CM-3500D colorimeter (Hunter Associates Laboratory, Stamford, CT, USA) in the CIELAB color space. CIE standard illuminant D65 and 10° standard observer were used as references. Three independent measurements of the a* (redness), b* (yellowness), and L* (lightness) parameters were taken from different areas of the flour and hydrated systems. The *E* index was calculated using the following equation:

(2) $$E=(L{*}^{2}+\;a{*}^{2}+\;b{*}^{2}{)}^{1/2}$$

The color difference (Δ*E*) was determined by comparing the results from the hydrated systems with those of the initial flours. The equipment was calibrated with a standard calibration CR-A43 white plate (a*/0.3156, b*/0.3319, and L*/93.80).

### 2.7. Determination of Reducing Sugars

Free reducing sugars were quantified using the 3,5-dinitrosalicylic acid (DNS) method [20]. Aqueous extracts of samples were mixed with DNS reagent and heated in a water bath at 100 °C for 15 min. After cooling, absorbance was measured at 540 nm. For total reducing sugars, samples underwent acid hydrolysis with HCl, followed by neutralization before applying the same DNS procedure. Glucose was used to build the calibration curve, within a concentration range of 0.25 to 2.0 mg/mL. The limit of quantification (LOQ) was estimated to be 0.2 mg/g. The samples were analyzed in duplicate and the results were expressed as mg of glucose equivalents/g of sample.

### 2.8. Determination of Acrylamide by LC-ESI-MS/MS

Acrylamide was determined in flours and hydrated systems using liquid chromatography coupled with electrospray ionization tandem mass spectrometry (LC-ESI-MS/MS), following the validated method described by Mesías and Morales [6]. An Agilent 1200 liquid chromatography system coupled to an Agilent triple quadrupole mass spectrometer (Agilent Technologies, Palo Alto, CA, USA) was employed for the analysis. Separation was performed on an Inertsil ODS-3 column (250 × 4.6 mm, 5 μm; GL Sciences Inc., Tokyo, Japan), maintained at 30 °C. The chromatographic run used isocratic elution with a mobile phase consisting of formic acid in water (0.2% *v*/*v*) at a flow rate of 0.4 mL/min. A 5 μL aliquot of each sample was injected. The mass spectrometer operated with electrospray ionization in positive mode. Under these conditions, acrylamide was eluted at 6.4 min. The spray needle voltage was set to 1.0 kV. Nitrogen served as the nebulizing gas at a flow rate of 12.0 L/min, and the ion source temperature was set to 350 °C. The instrument monitored Multiple Reaction Monitoring (MRM) transitions of *m*/*z* 72.1 > 55.1 for acrylamide and *m*/*z* 75.1 > 58.1 for (^13^C_3_)-acrylamide. Voltages for fragmentation were set to 50 V and 76 V for acrylamide and the internal standard, respectively. Corresponding collision energies were 11 V for *m*/*z* 72.0 > 55.1 and 8 V for *m*/*z* 75.0 > 58.1 transitions.

The accuracy of the results was assessed by the participation in the Food Analysis Performance Assessment Scheme (FAPAS) program. The latest results from the proficiency included coffee (test ID number 30117), crispbread (test ID number 30118), and potato crisps (test ID number 30133). These tests yielded z-scores of −0.1, 0.1, and −0.2, respectively, confirming the reliability of the results. The LOQ was estimated to be 15 µg/kg. The samples were analyzed in duplicate and the results were expressed as µg/kg sample.

### 2.9. Determination of Asparagine

The asparagine content in flours was analyzed using the same methodology as for acrylamide determination. In this case, the mass spectrometer operated with electrospray ionization in negative mode. Asparagine eluted at 2.5 min. The instrument monitored the MRM transition of *m*/*z* 131.04 > 114.00, with a voltage for fragmentation of 70 V and collision energy of 5 V. The samples were analyzed in duplicate and the results were expressed as µg/g sample.

### 2.10. Determination of Hydroxymethylfurfural (HMF) by HPLC-DAD

HMF concentrations in flours and hydrated samples were determined by HPLC-DAD, according to Mesías et al. [17]. The LOQ was estimated to be 0.6 µg/kg. The samples were analyzed in duplicate and the results were expressed as mg/kg sample.

### 2.11. Statistical Analysis

Statistical analyses were performed using a Statgraphics Centurion XV (Herndon, VA, USA). Results were expressed as mean ± standard deviation (SD). One-way ANOVA followed by Scheffé’s test were applied to determine differences between means. Differences were considered to be significant at *p* < 0.05. Relationships between the different parameters analyzed were evaluated by computing Pearson linear correlation coefficients at the *p* < 0.05 confidence level.

## 3. Results and Discussion

Sixteen flours made from cereals, pseudocereals, legumes, fruits, and roots were selected for this study: wheat, corn, durum wheat, oat, rice, rye, spelt, buckwheat, quinoa, teff, chickpea, lentils, soybean, chestnut, coconut, and cassava flours. The experimental design involved model systems adjusted to the maximum water-holding capacity of each flour type. Two separate trials were conducted in order to study the acrylamide formation during baking. In the first trial, dough was prepared with water and baked, mimicking a bread-like model system (water-hydrated system). This setup aimed to assess the impact of each flour’s raw composition, particularly their levels of reducing sugars and asparagine, on acrylamide formation. In the second trial, water was replaced by a glucose solution to enhance the Maillard reaction during baking (glucose-hydrated system), thereby simulating conditions to a biscuit-like model system. Additionally, both trials included a sodium chloride solution in the formulations, as salt plays a role in dough properties and can impact the formation of process contaminants in baked cereal products [21]. The thermal treatment conditions selected for this study (150 °C for 30 min) were based on previous literature evaluating acrylamide formation in model systems and cereal-based matrices under controlled laboratory conditions. These parameters have been commonly employed to simulate moderate- to high-temperature processing, such as baking or roasting, while still allowing for the quantification of acrylamide formation without reaching levels that could cause excessive sample degradation or charring [22,23,24]. The temperature of 150 °C represents an intermediate thermal condition commonly assessed in kinetic studies and reflects typical processing temperatures used in the production of bakery products, particularly in the crust area, where acrylamide formation is more likely to occur. A duration of 30 min ensures a sufficient reaction time for the Maillard reaction and acrylamide generation, while preserving the integrity of the model system. Moreover, this duration facilitates the standardization of the initial temperature drop that occurs upon opening the oven and introducing the samples. Thus, both temperature and time parameters were selected to ensure reproducible processing conditions and minimize uncontrolled experimental variability.

Flours were characterized by their moisture content, free reducing sugars, total reducing sugars, and asparagine content, as well as the water-holding capacity (Table 1). The moisture content of samples varied significantly, ranging from 5.7% in chestnut flour to 11.7% in buckwheat flour. An even greater variability was observed in WHC, which followed a descending order: fruits > legumes > pseudocereals > cereals > roots. While root and some cereal flours retained 65.2–76.3% of their weight, fruit-based flours such as coconut (424.6%) and chestnut (463.2%) exhibited considerably higher retention capacities. The remaining samples showed an intermediate WHC, ranging from 84% to 212%. The effective moisture retention capability of fruit-based flours compared with wheat flour has been previously reported [25], some of them being able to retain even 6.35 g of water/g of sample [26]. Among the analyzed flours, cassava flour did not contain detectable levels of either free or total reducing sugars. Quinoa flour showed the highest concentration of free reducing sugars (25.8 mg/g), while fruit-based flours exhibited the highest levels of total free reducing sugar content (217–235 mg/g). In contrast, oat and rice flours had the lowest concentrations of both free (0.7–1.6 mg/g) and total (5–10 mg/g) reducing sugars. Regarding asparagine content, rice flour had the lowest level (27 µg/g), whereas lentil flour showed the highest concentrations, reaching up to 1195 µg/g.

The pH values of the water-soluble fraction of flours fell within a similar range, varying from 6.1 in wheat flour to 7.0 in soybean flour (Table 2). After baking, the pH was either unaffected or slightly decreased in the systems containing water, with values ranging from 5.7 to 6.3, except for soybean flour, which dropped from 7.0 to 5.8. A more pronounced pH reduction was observed in glucose-hydrated systems, where values ranged from 4.9 to 6.1. The decrease in pH during the Maillard reaction is linked to the formation of acids, particularly short-chain carboxylic acids. This phenomenon occurs as various reaction pathways lead to acid generation, ultimately affecting the overall pH of the system [27]. Similar declines in flour pH after baking, especially in bread-crust-resembling model systems, have also been reported [28].

**Figure 1:**
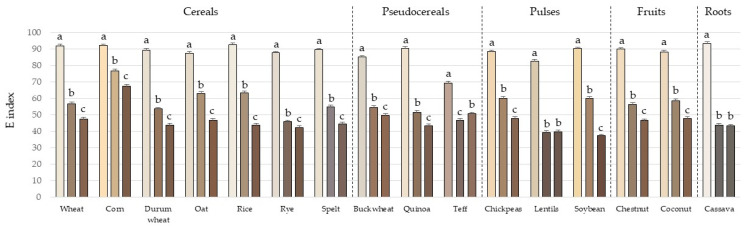
E index of flours (column 1), water-hydrated systems (column 2), and glucose-hydrated systems (column 3) for each cereal, pseudocereal, legume, fruit, and root flour. Results are mean ± SD. Different letters among the three columns for the same cereal, pseudocereal, legume, fruit, or root flour indicate significant differences (*p* < 0.05).

A color analysis using a colorimeter based on the CIELAB scale revealed significant differences among the samples, both in flours and in hydrated systems. The development of color is a key indicator of the progression of the Maillard reaction [29], which is characterized by a decrease in the L* parameter (indicating reduced brightness) and an increase in the a* parameter (Supplementary Table S1). Systems hydrated with water exhibited slight browning after baking due to their initial reducing sugar content (Table 1). However, the addition of glucose had a more pronounced effect on the final color, with significant differences (*p* < 0.05) observed between samples prepared with water and those containing glucose. In this regard, while L* values in flours ranged from 68.3 to 93.3, brightness decreased to a range of 38.3–74.4 in water-hydrated systems and further declined to 34.6–64.1 in glucose-hydrated systems. Similarly, the a* parameter increased from a minimum value of −0.6 to a maximum of 6.9 in systems with added glucose. Considering the L*, a*, and b* parameters, the overall color (E) was calculated for each sample, where lower values indicate increased browning (Figure 1) [30]. As shown in the graphs, all flours tended to darken after heating, with this effect being more pronounced in systems containing glucose. The most intense darkening was observed in lentil flour, while corn flour exhibited the lightest colors. Teff flour showed the least variation in color after baking with both water and glucose, likely due to its naturally darker hue in the unbaked state. When considering the initial flour color, spelt exhibited the least browning in both water- and glucose-hydrated systems, whereas lentils and coconut showed the highest browning in water- and glucose-hydrated systems, respectively.

Acrylamide was not detected in any of the flours. In the water-hydrated systems, only doughs made from wheat, rye, and coconut flours exhibited measurable acrylamide levels after baking, ranging from 23 to 61 µg/kg (Table 3), whereas the remaining displayed an acrylamide content below the LOQ (15 μg/kg). Acrylamide levels increased across all the glucose-hydrated systems as compared with the water-hydrated counterparts for most of the flour types. The exception was cassava flour, which showed no detectable acrylamide, and concentrations ranged from 20 µg/kg in corn flour to 154 µg/kg in lentil flour.

**Figure 2:**
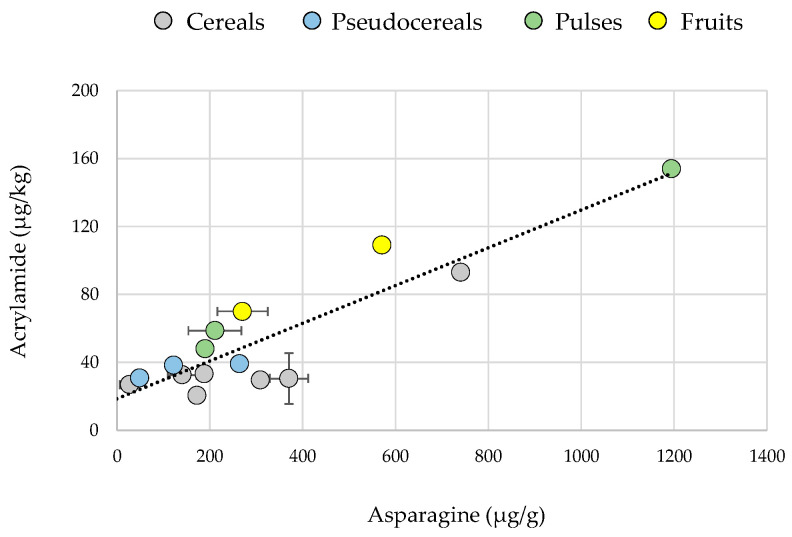
Correlation between asparagine content in flour and acrylamide levels in glucose-hydrated systems. Results are mean ± SD.

It is well-established that the free amino acid asparagine is the most relevant amino acid involved in acrylamide formation [31,32]. Moreover, in cereal products, asparagine serves as the primary limiting factor for acrylamide formation, playing a more critical role than the levels of reducing sugars [18]. Analyzing the flour compositions, wheat, rye, and coconut flours did not have the highest asparagine content but contained sufficient asparagine along with high levels of reducing sugars, promoting acrylamide formation. Conversely, while quinoa flour had the highest concentration of reducing sugars, its low asparagine content likely restricted acrylamide generation. Similarly, flours such as durum wheat, corn, spelt, oat, teff, lentil, chickpea, soybean, and chestnut had higher asparagine levels than wheat but contained reducing sugar levels below 10.2 mg/g—likely insufficient to react with asparagine and produce acrylamide. An exception was chestnut flour, which had both a higher asparagine content than wheat and reducing sugar levels comparable to rye. However, due to its exceptionally high water-holding capacity (463%), a substantial amount of water was retained before baking. This high moisture content may have reduced the Maillard reaction and, consequently, limited acrylamide formation. In glucose-hydrated systems, samples with the highest asparagine content resulted in the highest acrylamide levels, indicating that a greater sugar concentration was required in these formulations to enhance contaminant formation. A significant positive correlation was observed between asparagine levels in flour and acrylamide formation in glucose-hydrated systems (r = 0.8963, *p* < 0.0001) (Figure 2), underscoring the crucial role of this amino acid in acrylamide generation. However, it is worth emphasizing that a minimum amount of reducing sugars is crucial for this reaction, as acrylamide was negligible in systems without added sugar except for three specific flours discussed above. In this study, glucose was used as the reference reducing sugar; nevertheless, it should be noted that similar trends might have been observed with the inclusion of other sugars commonly used in food formulations, such as fructose or sucrose, although their concentrations and reactivity may differ.

Another factor to consider is that, under identical thermal treatment conditions, the asparagine content in flour is not the only determinant of acrylamide formation. The presence of other amino acids, and the ratio of asparagine to these amino acids, as well as the moisture content and pH of the hydrated systems, also influence acrylamide production [33,34].

Previous studies have reported higher acrylamide levels in cereal-based products formulated with rye compared to other cereals, such as corn, oat, and wheat, primarily due to the higher asparagine content in rye [28]. However, this trend is not consistent across all studies. For instance, Ciesarová et al. [33] reported higher acrylamide levels in oat flour heated at 140 °C for 15 min compared with rye flour, with concentrations of 1951 and 1833 µg/kg, respectively. Similarly, comparisons between cereal and legume flours have also yielded controversial results. While some studies suggest that incorporating legume flour into cereal-based products may increase acrylamide formation—possibly due to the differences in protein composition [35]—other research has reported that legume flours can enhance the nutritional profile of food products while reducing acrylamide formation [36,37]. Direct comparisons between results in the literature and those obtained in the present study are challenging, as acrylamide formation can vary depending on whether it is assessed in dry model systems, low-moisture systems, or food matrices. These differences are influenced by the distinct water-holding capacities of each flour. In the doughs evaluated in this study, the high moisture content—due to the minimal water loss during baking, as the tubes were sealed—likely limited the extent of the Maillard reaction and, consequently, acrylamide formation.

The role of amino acids other than asparagine in acrylamide formation must also be considered. These amino acids can either promote acrylamide formation or compete with asparagine in chemical reactions, potentially reducing its production, as reported in previous studies [38]. In this context, various studies have explored the addition of specific amino acids to reduce acrylamide in baked goods. Amrein et al. [39] found that small additions of L-glutamine, L-lysine, or glycine had little effect on acrylamide levels in gingerbread, whereas higher doses of glycine reduced acrylamide content by approximately two-thirds. Similarly, Capuano et al. [40] observed that adding glycine to bread crisps significantly decreased acrylamide levels, possibly due to glycine competing with reactive carbonyls or reacting directly with the acrylamide formed. Shen et al. [41] studied the effects of seven amino acids at various concentrations in white pan bread. They reported that asparagine increased acrylamide levels in the crust, while glycine and proline were effective in reducing it. L-cysteine also showed promising results, lowering acrylamide formation in gingerbread, crackers, and bread. In contrast to these findings, Ciesarová et al. [33] suggested that glutamine may act as a precursor to acrylamide in thermally treated flours, possibly due to its conversion into asparagine before heat treatment, in systems where enzymes such as aspartate transaminase and asparagine synthetase are active [42]. In addition to the type of amino acids contributed by different flours, the ratio of asparagine to other amino acids appears to play a crucial role in acrylamide formation during processing. For instance, when legumes are included in cereal-based formulations, this ratio may be altered, potentially influencing the Maillard reaction and subsequent acrylamide production [34]. These findings underscore the importance of carefully selecting ingredients and optimizing processing conditions to minimize acrylamide content while maintaining the nutritional benefits of incorporating legumes into cereal-based products.

In parallel with acrylamide determination, another chemical process contaminant, HMF, was also analyzed. HMF can be generated through both the Maillard reaction and caramelization and serves as an indicator of thermal damage [43]. Additionally, HMF can act as a precursor to acrylamide formation, making it an important factor to consider in the present study. HMF levels followed a pattern similar to acrylamide in the analyzed flours, as it was not detected in unheated samples but increased in hydrated systems containing water (0.1–11.7 mg/kg) and even more significantly in those formulated with glucose (64.1–232.3 mg/kg) (Table 3). This suggests that, in baked samples containing glucose, HMF formation is influenced by both the Maillard reaction and caramelization. Among the systems hydrated only with water, the lowest HMF levels (0.1 mg/kg) were found in rice, soybean, and cassava, while the highest levels were observed in quinoa. This trend may be attributed to the low reducing sugar content in rice, soybean, and cassava (<0.2–2.7 mg/g) compared to the high content in quinoa (25.8 mg/g) (Table 1). A significant correlation was observed between the reducing sugar content and HMF levels in water-hydrated systems (r = 0.8511, *p* < 0.0001). Notably, although oat had the lowest levels of reducing sugars, its lower water-holding capacity compared to rice and soybean resulted in reduced moisture in the system, which may have facilitated HMF formation by promoting the Maillard reaction. The addition of glucose had a strong impact on HMF formation, consistent with the findings of Gökmen et al. [44], who reported that glucose has a greater effect on HMF formation than other sugars such as sucrose. Under these conditions, the lowest HMF levels were observed in fruit and root flours, likely due to the high moisture content in systems with coconut and chestnut, as well as the lower sugar content in cassava flour. In contrast, the highest HMF levels were found in lentils, possibly influenced not only by their sugar content but also by their higher protein content compared to cereals [45]. No significant correlations were found between acrylamide and HMF levels across all systems evaluated in this study, suggesting that their formation flows down different pathways, influenced by distinct precursors and system conditions. Maybe the higher moisture content in the samples could explain the absence of this correlation, since it has been reported that HMF can be involved in the Maillard reaction under low moisture conditions at elevated temperatures with the following acrylamide formation in the presence of asparagine [46].

In general, lowering the pH increases the likelihood of HMF formation while decreasing acrylamide production in cereal-based foods during baking [44]. However, in this study, no significant correlations were observed between pH and the formation of either compound. Similarly, and contrary to expectations, HMF and acrylamide levels did not correlate with browning development after baking. This outcome may be explained by the water retained in the systems and the minimal water loss during baking, as the sealed tubes likely limited moisture evaporation. Consequently, this could have led to a reduced Maillard reaction and caramelization, resulting in a more controlled darkening of the baked samples.

### Limitations of the Study

One of the limitations of this study is that the thermal treatment was conducted in sealed tubes under constant temperature and time conditions, which differs from the dynamic and open nature of industrial baking processes. This controlled system was intentionally selected to minimize external variables—such as moisture loss and temperature gradients—and to allow for the standardized comparison of acrylamide formation across different flour types. While this setup does not replicate the full complexity of real baking environments, it provides a reproducible framework for isolating the effects of specific variables, such as flour composition and sugar addition.

Additionally, the present study focused solely on the quantification of free asparagine, given its well-established role as the main precursor of acrylamide in cereal-based products. Although the possible modulatory role of other amino acids has been discussed in the manuscript, their concentrations were not experimentally determined, which represents a valuable direction for future research.

Finally, the aim of this study was to characterize the intrinsic acrylamide-forming capacity of individual flour types without the influence of other ingredients. Since formulations in the food industry typically involve mixtures of wheat and alternative flours, future studies should investigate more representative flour blends to better reflect industrial practices.

## 4. Conclusions

This study highlights the significant variability in acrylamide formation among different types of flours used as alternatives to traditional wheat flour, including cereal, pseudocereal, legume, fruit, and root flours. While replacing wheat with alternative flours can enhance the nutritional and sensory qualities of baked goods, it may also pose a potential risk due to acrylamide formation, which is influenced by the composition and physicochemical properties of the flours. The outcomes indicate that the addition of glucose in systems composed of flours other than wheat can significantly enhance acrylamide formation, with increases of up to 4.7-fold. It is noteworthy to highlight that the incorporation of legume flours, particularly lentils, or fruit-based flours such as coconut flour, as substitutes for wheat in cereal-based food formulations containing sugars should be evaluated from the perspective of a potential increase in chemical risk. Acrylamide levels rose from 33 µg/kg in wheat flour to 154 µg/kg in lentil flour and 109 µg/kg in coconut flour. For such formulations, implementing additional mitigation strategies, including enzymatic treatments or pH adjustments, may help reduce acrylamide levels and improve the overall safety profile of the final products. Hydroxymethylfurfural (HMF), evaluated both as an indicator of thermal damage and as a toxicological concern, was also formed during baking. Its levels followed a similar trend to acrylamide, with lentil flour again standing out as the greatest contributor, reaching 232.3 mg/kg. These findings underscore the importance of thoroughly assessing the potential formation of toxicological compounds when developing cereal-based products with non-traditional flours. Such evaluations are essential in order to ensure that the nutritional improvements do not compromise food safety. Future research should explore acrylamide formation over time to gain further insights into the dynamics of its formation during baking.

## Figures and Tables

**Table 1:** Characterization of flours in terms of moisture content (%), water-holding capacity (WHC) (%), free reducing sugars (mg/g), total reducing sugars (mg/g), and asparagine (µg/g).

Samples	Moisture	WHC	Free Reducing Sugars	Total Reducing Sugars	Asparagine
Cereals					
Wheat	10.3 ± 0.0 g	83.6 ± 3.4 b	17.1 ± 0.3 l	30.0 ± 0.3 bc	141 ± 31 ab
Corn	10.8 ± 0.1 h	141.3 ± 0.4 g	7.5 ± 0.1 g	26.0 ± 0.5 b	172 ± 14 ab
Durum wheat	10.6 ± 0.0 gh	76.3 ± 0.8 ab	10.1 ± 0.2 h	30.2 ± 1.1 b	188 ± 9 ab
Oat	9.9 ± 0.1 f	97.1 ± 0.6 c	0.7 ± 0.0 a	11.0 ± 0.2 a	309 ± 21 abc
Rice	10.8 ± 0.1 f	107.5 ± 0.4 fg	1.6 ± 0.0 j	6.7 ± 0.1 a	27 ± 2 d
Rye	10.0 ± 0.1 h	136.6 ± 0.8 cd	13.5 ± 0.2 b	95.5 ± 2.1 f	741 ± 41 a
Spelt	10.5 ± 0.0 g	65.2 ± 0.6 a	5.9 ± 0.2 e	36.6 ± 0.5 c	370 ± 0 bc
Pseudocereals					
Buckwheat	11.7 ± 0.0 i	134.0 ± 0.0 fg	4.0 ± 0.4 d	25.1 ± 0.2 b	122 ± 14 ab
Quinoa	9.6 ± 0.0 e	108.9 ± 0.5 cd	25.8 ± 0.3 m	48.8 ± 1.8 d	49 ± 4 a
Teff	9.9 ± 0.0 f	116.6 ± 0.3 de	6.6 ± 0.1 e	26.7 ± 0.4 b	264 ± 4 ab
Legumes					
Chickpea	8.9 ± 0.0 d	106.4 ± 0.6 cd	7.5 ± 0.2 g	74.3 ± 1.1 e	211 ± 12 ab
Lentils	9.9± 0.0 f	123.9 ± 1.3 ef	6.8 ± 0.0 f	72.3 ± 2.9 e	1195 ± 57 e
Soybean	6.7 ± 0.0 b	212.8 ± 2.9 h	2.7 ± 0.0 c	124.7 ± 2.5 g	190 ± 0 ab
Fruits					
Chestnut	5.7 ± 0.0 a	463.2 ± 1.6 j	11.3 ± 0.1 i	229.0 ± 4.2 h	270 ± 2 ab
Coconut	7.0 ± 0.0 c	424.6 ± 8.3 i	16.1 ± 0.1 k	251.1 ± 1.9 i	571 ± 71 cd
Roots					
Cassava	10.8 ± 0.0 h	75.4 ± 1.6 ab	<LOQ	<LOQ	<LOQ

Results are mean ± SD. LOQ: Limit of quantification. Different letters in the same column mean significant differences (*p* < 0.05).

**Table 2:** pH of flours and systems hydrated with water or glucose.

Samples	Flour	Water	Glucose
Cereals			
Wheat	6.1 ± 0.0 a	6.2 ± 0.0 bcdef	5.6 ± 0.0 e
Corn	6.2 ± 0.0 ab	6.1 ± 0.0 abcde	5.4 ± 0.0 cde
Durum wheat	6.7 ± 0.0 e	6.2 ± 0.0 cdef	5.4 ± 0.1 de
Oat	6.2 ± 0.0 ab	6.3 ±4 0.2 f	5.5 ± 0.0 e
Rice	6.6 ± 0.0 cde	6.2 ± 0.0 cdef	5.2 ± 0.0 bc
Rye	6.5 ± 0.0 de	6.3 ± 0.0 ef	6.1 ± 0.0 f
Spelt	6.2 ± 0.0 ab	6.3 ± 0.1 def	4.9 ± 0.0 ab
Pseudocereals			
Buckwheat	6.7 ± 0.0 e	5.9 ± 0.0 abcd	5.3 ± 0.1 cd
Quinoa	6.5 ± 0.0 cde	6.0 ± 0.3 abc	5.4 ± 0.1 de
Teff	6.7 ± 0.0 e	6.3 ± 0.0 ef	5.4 ± 0.0 cde
Legumes			
Chickpea	6.3 ± 0.0 abc	6.0 ± 0.3 abcd	4.9 ± 0.0 ab
Lentils	6.3 ± 0.0 abc	5.7 ± 0.2 abc	5.4 ± 0.0 cde
Soybean	7.0 ± 0.0 f	5.8 ± 0.1 abc	5.4 ± 0.0 cde
Fruits			
Chestnut	6.4 ± 0.0 bcd	5.7 ± 0.1 a	4.9 ± 0.1 a
Coconut	6.3 ± 0.0 abc	5.7 ± 0.1 ab	5.3 ± 0.1 cd
Roots			
Cassava	6.6 ± 0.1 de	5.8 ± 0.2 def	5.2 ± 0.0 cd

Results are mean ± SD. LOQ: Limit of quantification. Different letters in the same column mean significant differences (*p* < 0.05).

**Table 3:** Acrylamide (µg/kg) and HMF (mg/kg) levels in water- and glucose-hydrated systems.

	Acrylamide		HMF	
Samples	Water	Glucose	Water	Glucose
Cereals				
Wheat	23 ± 1 a	33 ± 1 abc	2.5 ± 0.2 bcde	121.9 ± 1.01 bcd
Corn	<LOQ	20 ± 1 a	1.0 ± 0.1 ab	94.8 ± 2.4 abc
Durum wheat	<LOQ	33 ± 0 abc	1.9 ± 0.1 abcd	99.3 ± 1.2 abc
Oat	<LOQ	30 ± 3 ab	0.5 ± 0.0 ab	126.5 ± 9.4 bcde
Rice	<LOQ	27 ± 1 ab	0.1 ± 0.0 a	80.3 ± 4.4 ab
Rye	37 ± 4 a	93 ± 15 ef	7.3 ± 0.5 f	139.5 ± 9.2 cdef
Spelt	<LOQ	31 ± 0 ab	1.6 ± 0.2 abc	151.9 ± 0.3 defg
Pseudocereals				
Buckwheat	<LOQ	38 ± 2 abc	1.2 ± 0.0 ab	139.7 ± 8.9 cdef
Quinoa	<LOQ	31 ± 2 ab	11.7 ± 1.0 g	176.6 ± 9.7 fg
Teff	<LOQ	39 ± 2 abc	3.9 ± 0.2 de	180.1 ± 12.4 fg
Legumes				
Chickpea	<LOQ	59 ± 4 cd	1.5 ± 0.2 abc	193.4 ± 6.0 gh
Lentils	<LOQ	154 ± 4 g	1.6 ± 0.1 abc	232.3 ± 16.9 h
Soybean	<LOQ	48 ± 1 bcd	0.1 ± 0.0 a	170.5 ± 9.2 efg
Fruits				
Chestnut	<LOQ	70 ± 3 de	3.6 ± 0.5 cde	67.7 ± 0.6 a
Coconut	61 ± 8 b	109 ± 4 f	4.4 ± 0.6 e	64.1 ± 2.3 a
Roots				
Cassava	<LOQ	<LOQ	0.1 ± 0.0 a	69.0 ± 4.5 a

Results are mean ± standard deviation. LOQ: Limit of quantification. Different letters in the same column mean significant differences (*p* < 0.05).

## Data Availability

The original contributions presented in the study are included in the article; further inquiries can be directed to the corresponding author.

## Supplementary Materials

The following supporting information can be downloaded at: https://www.mdpi.com/article/10.3390/foods14091597/s1, Table S1: CIELAB color parameters in flours and baked doughs model systems formulated with water or glucose.

## Abbreviations

The following abbreviations are used in this manuscript:

AOAC	Association of Official Analytical Chemists
EFSA	European Food Safety Authority
FAPAS	Food Analysis Performance Assessment Scheme
HMF	Hydroxymethylfurfural
HPLC-DAD	High-performance liquid chromatography with diode-array detection
LC-ESI-MS/MS	Liquid chromatography coupled with electrospray ionization tandem mass spectrometry
SD	Standard deviation
WHC	Water-holding capacity
