# Supplementary material for: Acrylamide- and Hydroxymethylfurfural-Forming Capacity of Alternative Flours in Heated Dough Systems
Source: Foods. 2025 Apr 30;14(9):1597. doi: 10.3390/foods14091597 (PMC12071718; doi:10.3390/foods14091597)
Supplement: Supplementary file 1 [file foods-14-01597-s001.zip › foods-3609078-supplementary.pdf]

Supplementary material

Table S1. CIELAB color parameters in flours and baked doughs model systems formulated with water or glucose.

	Flour	Water	Glucose	Flour	Water	Glucose
	Wheat			Corn		
L*	91.4 ± 0.6 hi	54.7 ± 0.1 efgh	44.4 ± 0.6 cde	89.6 ± 0.4 fgh	73.4 ± 0.6 j	64.1 ± 1.6 f
a*	0.0 ± 0.1 bc	3.5 ± 0.4 cdef	6.7 ± 0.6 cdefg	1.7 ± 0.1 fab	1.9 ± 0.4	4.9 ± 0.7 bcd
b*	8.4 ± 0.2 cd	14.5 ± 1.5 def	15.5 ± 2.0 cdef	21.0 ± 0.1 h	22.1 ± 1.6 g	20.4 ± 2.3 f
	Durum wheat			Oat		
L*	88.8 ± 1.1 efg	50.3 ± 2.7 cdef	41.0 ± 3.0 bcd	87.0 ± 0.1 cde	60.5 ± 0.8 hi	42.2 ± 1.3 bcd
a*	−0.0 ± 0.1 b	6.0 ± 0.9 hi	6.9 ± 0.9 defg	0.3 ± 0.2 bc	3.1 ± 0.6 bcd	8.7 ± 1.1 g
b*	8.6 ± 0.3 cd	17.3 ± 1.4 ef	13.5 ± 1.8 cde	9.2 ± 0.6 de	17.0 ± 1.4 ef	17.5 ± 2.0 ef
	Rice			Rye		
L*	92.3 ± 0.4 i	61.3 ± 0.9 i	40.9 ± 0.4 bcd	87.2 ± 0.3 def	44.6 ± 1.4 bc	39.5 ± 1.3 abc
a*	−0.6 ± 0.0 a	2.7 ± 0.6 bc	7.6 ± 0.9 efg	0.1 ± 0.0 bc	3.8 ± 0.6 cdefg	6.5 ± 0.2 bcdef
b*	6.8 ± 0.2 b	15.1 ± 2.1 def	13.6 ± 2.8 cde	7.7 ± 0.1 bc	9.5 ± 1.3 bc	13.3 ± 1.2 cde
	Spelt			Buckwheat		
L*	89.0 ± 0.6 efgh	53.4 ± 2.6 efg	43.4 ± 2.4 bcde	84.7 ± 0.7 c	51.5 ± 1.1 defg	46.2 ± 2.1 de
a*	0.1 ± 0.1 bc	3.6 ± 0.5 cdef	4.7 ± 0.6 bc	0.0 ± 0.2 b	6.9 ± 0.3 i	8.2 ± 0.3 fg
b*	8.9 ± 0.4 cd	12.4 ± 1.9 cd	8.0 ± 1.4 ab	6.9 ± 0.7 b	16.4 ± 0.8 def	16.2 ± 1.0 cdef
	Quinoa			Teff		
L*	89.6 ± 0.2 gh	49.2 ± 1.0 bcde	41.2 ± 3.0 bcd	68.3 ± 0.8 a	45.9 ± 1.0 bcd	48.7 ± 2.0 e
a*	−0.1 ± 0.0 ab	5.0 ± 0.6 fgh	5.9 ± 0.6 bcde	4.3 ± 0.2 g	4.6 ± 0.2 defgh	6.1 ± 0.6 bcde
b*	11.4 ± 0.1 f	14.1 ± 1.3 de	12.1 ± 0.7 bcd	10.3 ± 0.3 ef	7.3 ± 1.0 b	12.2 ± 1.6 bcd
	Chickpeas			Lentils		
L*	85.9 ± 0.6 cd	57.0 ± 13 ghi	44.6 ± 1.7 cde	81.2 ± 0.4 b	38.3 ± 2.8 a	38.6 ± 2.4 ab
a*	1.4 ± 0.1 ef	4.5 ± 0.7 defgh	7.1 ± 0.9 efg	−0.1 ± 0.1 b	4.7 ± 0.8 efgh	4.6 ± 0.6 b
b*	20.4 ± 0.1 h	18.3 ± 1.5 fg	15.1 ± 2.3 cdef	15.2 ± 0.1 g	7.3 ± 0.9 b	7.4 ± 2.1 ab
	Soy			Chestnut		
L*	86.3 ± 0.4 cd	56.8 ± 0.8 ghi	34.6 ± 1.4 a	88.3 ± 0.5 defg	54.2 ± 5.8 efg	42.9 ± 0.8 bcd
a*	0.2 ± 0.1 bc	5.2 ± 0.2 gh	7.8 ± 0.9 efg	1.2 ± 0.1 e	2.8 ± 0.2 bc	7.5 ± 0.6 efg
b*	26.0 ± 0.3 i	18.2 ± 1.5 fg	11.3 ± 2.0 bc	16.2 ± 0.1 g	14.7 ± 1.8 def	16.3 ± 1.3 cdef
	Coconut			Cassava		
L*	86.7 ± 0.1 cde	56.4 ± 0.8 fghi	44.0 ± 0.5 bcde	93.3 ± 0.4 i	43.7 ± 1.4 ab	43.0 ± 2.3 bcd
a*	0.9 ± 0.2 de	3.4 ± 0.5 bcde	7.2 ± 0.5 efg	0.6 ± 0.0 cd	0.7 ± 0.1 a	0.7 ± 0.1 a
b*	15.7 ± 0.4 g	15.2 ± 0.5 def	16.8 ± 0.7 def	3.7 ± 0.1 a	2.3 ± 0.3 a	4.3 ± 0.5 a

Results are mean ± standard deviation. LOQ: Limit of quantification. Different letters in the same column mean significant differences ($p < 0.05$).
